# Cultural development and validation of a group-based Somatic Experiencing® intervention for Indonesian women survivors of sexual assault with PTSD symptoms: a mixed-methods study

**DOI:** 10.3389/fpsyg.2026.1751747

**Published:** 2026-05-21

**Authors:** Ligina Ayudia, Fredrick Dermawan Purba, Annemarie Samuels, Aulia Iskandarsyah

**Affiliations:** 1 Postgraduate Program in Psychology, Faculty of Psychology, Universitas Padjadjaran, Bandung, Indonesia; 2 Department of Clinical and Health Psychology, Faculty of Psychology, Universitas Padjadjaran, Bandung, Indonesia; 3 Center for Psychological Innovation and Research, Faculty of Psychology, Universitas Padjadjaran, Bandung, Indonesia; 4 Institute of Cultural Anthropology and Development Sociology, Leiden University, Leiden, Netherlands

**Keywords:** cultural adaptation, Indonesia, intervention development, low-resource settings, PTSD, sexual assault, Somatic Experiencing®, trauma therapy

## Abstract

**Background:**

Sexual assault affects approximately 35.6% of women globally. In Indonesia, one in three women aged 15–64 has experienced physical and/or sexual violence, often resulting in post-traumatic stress disorder (PTSD). While evidence-based PTSD treatments exist, they often face limitations in cultural relevance and applicability in low-resource settings. Somatic Experiencing®, a body-oriented trauma therapy, has shown promise across diverse populations, yet remains underexplored among Indonesian women survivors.

**Objective:**

This study aimed to develop a culturally adapted, group-based SE® intervention tailored for Indonesian women survivors of sexual assault.

**Method:**

A mixed-method, cross-sectional design was employed for this Phase 1: Development and Cultural Validation Study. The development process followed the Generalized Model for Program Planning, encompassing needs assessment, goal setting, intervention design, module creation, translation, and cultural validation using the Cultural Relevance Questionnaire (CRQ). Expert reviewers provided both quantitative and qualitative feedback to refine linguistic, conceptual, and cultural fit.

**Results:**

The adapted intervention demonstrated high levels of cultural relevance: linguistic (*M* = 4.67; 93%), conceptual (*M* = 4.46; 93%), and cultural (*M* = 4.60; 90%). Expert reviewers supported that the intervention aligned with key Indonesian cultural values, including collectivism, emotional restraint, and indirect communication.

**Conclusion:**

This study represents Phase 1 of a three-phase research program to establish a culturally grounded, group-based Somatic Experiencing® intervention for Indonesian women survivors of sexual assault. This Phase 1 study focuses exclusively on intervention development and expert-based cultural validation. With Phase 2 (feasibility and pilot) successfully completed and the Phase 3 RCT protocol published, the program provides a rigorous, sequential foundation for evaluating the clinical effectiveness of SE in low-resource, collectivist contexts. Feasibility, participant acceptability, and clinical effectiveness are addressed in subsequent phases of the research program.

## 1 Introduction

Sexual assault affects 35.6% of women globally (World Health Organization, 2013). In Indonesia, 1 in 3 women aged 15–64 has reported experiencing physical and/or sexual assault during their lifetime, either by a partner or a non-partner (Badan Pusat Statistik, 2017). A 2016 online survey by Lentera Sintas Indonesia (2016) similarly suggested a high burden with 46.7% of 25,213 participants had experienced some form of sexual assault (Lentera Sintas Indonesia, 2016). Additionally, Perempuan (2021) recorded reported 955 cases of sexual assault in public and neighborhood spaces during 2020 (Perempuan, 2021), which reflects documented reports rather than population prevalence.

Sexual assault refers to any attempt to obtain sexual acts through coercion or force such as rape by an intimate partner or strangers (Krug et al., 2002). Epidemiological studies have shown that sexual assault is the trauma with the highest risk of resulting in Post-Traumatic Stress Disorder (PTSD) (Kessler et al., 1995). PTSD symptomatology spans intrusion, avoidance, negative alterations in mood and cognition, and arousal/reactivity (American Psychiatric Association, 2022). Common clinical features in survivors also include hyperarousal, dissociation, emotional immobility, and constriction- responses viewed as incomplete defensive reactions to trauma (Levine, 1977; Van der Kolk, 1994). When unresolved, these symptoms can lead to long-term dysregulation of the hypothalamic- pituitary adrenal (HPA) axis, interpersonal trauma, demoralization, and stigma (Payne et al., 2015; Yehuda et al., 2015).

Despite increased visibility of sexual violence and greater efforts to access support services, many survivors still face significant barriers to trauma-informed care. Recent literature indicates an increase in the number of women pursuing legal, medical, and psychological support for sexual trauma (Baert et al., 2021). However, many survivors with PTSD still face inadequate care and treatment barriers, often leading to poor compliance (Bach et al., 2021; Baert et al., 2021). Evidence-based therapies such as Eye Movement Desensitization and Reprocessing (EMDR), Cognitive Behavioral Therapy (CBT), and Prolonged Exposure (PE) have demonstrated efficacy in reducing PTSD symptoms (Bisson et al., 2013), although dropout rates in trauma-focused therapies remain a persistent challenge, with meta-analytic evidence indicating that approximately 18–25% of patients discontinue treatment prematurely in exposure-based and cognitively demanding modalities (Imel et al., 2013; Lewis et al., 2020), High dropout has been linked to difficulties tolerating trauma exposure and autonomic dysregulation (Corrigan and Hull, 2015; Lewis et al., 2020; Watts et al., 2013). Moreover, cognitive impairments resulting from trauma may also reduce the effectiveness of these therapies, leaving many treatments processes incomplete (Van der Kolk, 1994).

In contrast to these limitations, Somatic Experiencing® offers several features that may directly address these barriers. First, its emphasis on body awareness and non-verbal processing aligns with the tendency for somatic expression of distress, reducing reliance on verbal trauma narratives. Second, SE can be delivered in a gradual, titrated manner that minimizes emotional overwhelm, potentially lowering dropout risk. Third, its adaptability to group-based formats enhances scalability and accessibility in low-resource settings. Finally, the use of choice-based, non-directive language may better align with cultural norms of emotional restraint and indirect communication, thereby improving engagement among Indonesian survivors.

Taken together, these features directly address the previously identified barriers: reduced reliance on verbal processing aligns with somatic idioms of distress, the titrated approach may improve tolerability and reduce dropout, and the group-based format enhances scalability in low-resource settings.

These limitations are particularly acute in low-resource contexts like Indonesia, where access to mental health care is scarce and therapies often fail to align with sociocultural realities (Perempuan, 2021). In practice, CBT and EMDR frequently struggle to reach and effectively benefit Indonesian women survivors for three recurring reasons: (1) structural access barriers (limited specialist availability outside urban centers, travel, and cost burdens, and stigma); (2) a modality culture misfit, as services remain talk-heavy while many survivors express distress via somatic idioms (e.g., headaches, abdominal pain, fatigue etc.), a pattern widely documented in cross-cultural trauma literature, particularly in Asian and low-resource settings (Hinton and Kirmayer 2017; Hinton and Lewis-Fernández, 2011; Miller and Rasmussen, 2014); and (3) delivery demands (trained, supervision, privacy, and regular multi-session schedule) that are difficult to guarantee in routine services, contributing to treatment discontinuation and reduced effectiveness (Ayudia et al., 2025a,b; Hinton and Lewis-Fernández, 2011).

In response to these gaps, researchers and clinicians have turned to somatic, bottom-up approaches that directly engage the body’s physiological stress responses. Somatic Experiencing® is a biopsychological model developed by Peter Levine and Frederick, 1997. The term “*soma*” refers to the living, sensing body, while “*experiencing*” emphasised being present in the here and now. SE® facilitates completion of defensive body reaction (biological survival responses), stabilizes dysregulated autonomic nervous system activity, and enhances resilience through titrated exposure to body-based cues of trauma (Levine and Frederick, 1997; Neslihan, 2021). SE supports trauma resolution without requiring explicit narrative processing (Kuhfuß et al., 2022; Porges, 2022).

In addition, body-oriented approaches such as Somatic Experiencing® may be particularly relevant in non-Western and collectivist contexts, where psychological distress is frequently expressed through somatic symptoms rather than verbal emotional disclosure. Cross-cultural research has consistently demonstrated that trauma survivors in Asian settings often present with somatic idioms of distress, which may not be adequately addressed by cognitively oriented therapies (Devon E Hinton and Lewis-Fernández, 2011; Devon E. Hinton and Patel, 2017). This non-verbal and body-centered orientation may also reduce avoidance and improve engagement among survivors with high levels of shame or dissociation (Payne et al., 2015; Van der Kolk, 1994).

A systematic review and meta-analysis synthesized 16 empirical studies evaluating SE across diverse trauma-exposed populations (Kuhfuß et al., 2021). The pooled analysis documented moderate effect sizes for reductions in PTSD (Hedges’ *g* ≈ 0.58) and somatic symptoms (*g* ≈ 0.61), with additional gains in emotional regulation and perceived wellbeing. Some studies indicated large pre–post improvements in depression (Cohen’s *d* = 0.7–1.08), and symptom remission rates between 44–90% after 3–20 SE sessions. Nonetheless, methodological limitations were prominent: only a minority were randomized controlled trials; most studies suffered from small convenience samples, non-standardized outcome measures, and high heterogeneity ( $I^{2}$ >70%). Few studies included long-term follow-up or monitored therapist fidelity, constraining the generalizability and quality of evidence (Almeida et al., 2019; Brom et al., 2017; Sinha, 2024). Collectively, these findings highlight SE as a promising but still developing intervention requiring further rigorous, context-specific evaluation. Although dropout rates in SE studies are not consistently reported, preliminary findings suggest good acceptability and retention in small-scale studies (Kuhfuß et al., 2021).

While emerging evidence from scoping reviews indicates that Somatic Experiencing® (SE®) shows preliminary positive effects on PTSD-related symptoms, the literature highlights persistent limitations including mixed study quality, small samples, few RCTs, and heterogeneous outcomes. For instance, only two high-quality RCTs exist, with overall evidence urging stronger experimental designs despite promising results in symptom reduction. Proposed mechanisms like autonomic regulation and polyvagal theory frameworks remain theoretically grounded but lack consistent robust empirical support, positioning SE® as a developing trauma therapy (Kuhfuß et al., 2021). High risk of bias in non-RCTs, limited follow-ups, and variable measures undermine generalizability. Mechanisms (e.g., autonomic nervous system regulation via interoception) are proposed neurophysiologically but require direct validation beyond theory. SE® complements established therapies but merits further rigorous testing. Together, these findings suggest that SE is a promising but still developing intervention, warranting further rigorous and context-specific investigation.

Mechanistically, SE operates via bottom-up regulation, prioritizing bodily awareness through interoception (visceral sensation), titration (gradual, paced engagement with distress), and pendulation (oscillation between distress/resourcing), with the aim of re-stabilizing autonomic arousal. This approach aligns with polyvagal theory (Porges, 2022), suggesting trauma recovery depends on restoring adaptive vagal regulation to foster safety and social engagement.

Although most studies focus on individual SE formats, recent research explores group-based SE, which may offer added benefits: normalization, social buffering, and cost-effectiveness, highly relevant in Indonesia where social support is a protective factor for survivors (Nickerson et al., 2011; Ozer et al., 2003; Van der Kolk, 1994).

This study does not evaluate clinical effectiveness but instead focuses on developing and culturally validating a group-based Somatic Experiencing® intervention for Indonesian survivors of sexual assault, ensuring linguistic, conceptual, and experiential fit. This step represents a critical precursor to subsequent feasibility and effectiveness trials in LMIC settings.

This study is novel in three key ways: (1) it systematically develops a culturally adapted Somatic Experiencing® intervention for Indonesian women survivors of sexual assault, directly addressing the gap between high somatic distress and the lack of body-oriented interventions in LMICs; (2) it applies a group-based format designed for scalability in low-resource, collectivist settings; and (3) it integrates mixed-method cultural validation to ensure linguistic, conceptual, and contextual fit prior to effectiveness testing, thereby establishing a contextually grounded foundation for subsequent evaluation.

## 2 Methods

### 2.1 Study overview

This study represents phase 1: Development and cultural validation study of three-phase research program to develop and evaluate a culturally adapted, group - based Somatic Experiencing® intervention for Indonesian women survivors of sexual assault with PTSD symptoms. Phase 1 focused on the development, translation, and cultural validation of the intervention; subsequent phases (Phase 2: Feasibility and Pilot and Phase 3: Effectiveness Evaluation) are summarised in Supplementary Table S1 to illustrate program continuity.

The needs assessment was conducted with clinicians rather than survivors themselves. This decision was guided by ethical and safety considerations appropriate to early-stage intervention development with a highly vulnerable population. Clinicians were selected due to their extensive experience working with survivors, allowing them to provide aggregated insights across multiple cases. However, this approach may limit ecological validity and does not substitute for direct survivor perspectives. Future phases of the research program will incorporate survivor input, particularly during feasibility and pilot testing, to further refine the intervention and ensure its relevance and acceptability.

### 2.2 Design and approach

A mixed-method, cross-sectional design was employed, integrating qualitative need assessment and cultural validation. Intervention development was guided by the Generalized Model for Program Planning (McKenzie et al., 2021), which included need assessment, goal setting, intervention design, module drafting, translation, and cultural validation using the Cultural Relevance Questionnaire (CRQ). The process also integrated structured feedback from Indonesian psychologists and Somatic Experiencing® practitioners/experts. Reporting adhered to best-practice guidance for transparent methodological description. In keeping expectations for development studies, this phase did not test clinical outcomes; outcome evaluation (e.g., PCL-5, CD-RISC-25, WHOQOL-BREF) is planned for subsequent feasibility/pilot trials (Almasyhur and Nasrun, 2021; Ayudia et al., 2025a, 2025b; Ch Salim et al., 2007).

### 2.3 Setting and participant characteristics

#### 2.3.1 Needs-assessment interviews

Participants were licensed Indonesian clinical psychologist currently providing services to women survivors of sexual assault in health centers, NGOs, and private practice across Jakarta, West Java, Central Java, and Yogyakarta. Inclusion criteria: (1) ≥ 2 years of post-licensure clinical experience; (2) ≥ 12 months’ experience treating adult women survivors; (3) fluency in Bahasa Indonesia; (4) willingness to be audio-recorded. Exclusion criterion: current role limited exclusively to administrative/managerial duties. Sampling strategy: purposive and snowball sampling via professional networks and survivor-serving organizations. Twelve psychologists were recruited (10 female, 2 males; mean clinical experience ≈ 7 to 10 years). Data saturation was monitored throughout; topic saturation was reached at interview #10, with two additional interviews conducted to confirm stability of themes.

#### 2.3.2 Data collection: needs assessment

Method: semi-structured, in-depth interviews conducted by a clinical psychologist-researcher trained in qualitative methods. Mode & duration: in person or secure video call; 60–90 min each; audio-recorded. Language: Bahasa Indonesia.

Interview guide:

1. “*Ceritakan pengalaman Anda menangani penyintas kekerasan seksual—keluhan apa yang paling sering muncul*?” (presenting problems: overt/covert symptoms; somatic complaints)
2. “*Hambatan terbesar apa dalam terapi (klinis, keluarga, budaya, hukum)?”* (care barriers; drop-out)
3. “*Pendekatan terapi apa yang biasa Anda gunakan dan apa keterbatasannya*?” (current practices; perceived gaps)
4. “*Keterampilan regulasi tubuh/emosi apa yang paling realistis diajarkan di kelompok*?” (priorities for skills)
5. “*Apa saran Anda untuk struktur sesi, durasi, dan hal lain yang berkaitan dengan terapi*?” (logistics; safety).

In addition to interviews, the researcher conducted targeted literature search to sharpen the need assessment and to inform culturally sensitive intervention design. Searches were run in in PubMed, Google Scholar, GARUDA, and Neliti (2010–2025) using terms related to Indonesia/LMICs, sexual assault/trauma survivors, group and somatic interventions (e.g., Somatic Experiencing®), and cultural values. Peer-reviewed reviews and empirical studies in Indonesian or English that addressed service context, cultural values, and clinical language were included; opinion pieces and non-scholarly reports were excluded. Findings from this literature were triangulated with interview data and used to refine facilitator language, session structure, and safety protocols.

### 2.4 Data analysis

Interviews were transcribed verbatim in Bahasa Indonesia and de-identified. A thematic analysis approach combining inductive and deductive strategies was employed. First, an initial codebook was developed based on the interview guide and a subset of early transcripts. Second, approximately 25–30% of the transcripts were double-coded by an independent second analyst to enhance analytical rigor. Third, consensus meetings were conducted to resolve coding discrepancies and iteratively refine the coding framework and emerging themes. Fourth, themes were systematically organized into matrices linking thematic findings with illustrative quotations and corresponding practice implications. Data management and organize were supported using NVivo software.

All qualitative quotations reported in the results were translated to English using a meaning-preserving approach and checked by a researcher.

#### 2.4.1 Translation and adaptation workflow

To ensure linguistic and conceptual fidelity while maintaining cultural resonance, we followed a four-step bilingual workflow. First, module drafting in English by the research team and SE experts. Second, forward translation into Bahasa Indonesia by researcher. Third, an expert panel reconciliation (2 SE® practitioners + 3 clinician) to harmonize technical terms (e.g., grounding → *membumi*, felt sense → *merasakan sensasi*, pendulation → *pendulasi/bergerak bolak-balik* with plain-language gloss). Finaly, cognitive debriefing with Indonesian clinicians to test clarity/acceptability; revisions prioritized gentle, choice-based phrasing (e.g., “kalau kamu mau, kamu boleh mencoba ini”) and neutral prompts avoiding potentially triggering references to “body” (“Apa yang kamu perhatikan di dalam dirimu atau di sekitarmu?”).

### 2.5 Co-design and expert consultation

Eight SE® experts (four study experts from SE International Institute and four Indonesian SE® practitioners) collaborated with the researcher to refine module. The researcher first distributed the draft to all experts, who then provided detailed written feedback. This process was followed by a series of consensus meetings to further discuss and revise the content. Revisions focused on clarifying session aims and flow, strengthening safety language, refining choice architecture and trigger-mitigation options, and ensuring the use of culturally consonant examples, imagery, and group agreements. In addition, to protect participants in any subsequent group implementations, the experts emphasised the need for clearly specified safety protocols within the module. Experts were selected based on predefined criteria, including a minimum of 3 years of SE® practice and prior experience in group-based trauma work.

### 2.6 Cultural validation

The Indonesian-language modules underwent cultural validation using the Cultural Relevance Questionnaire (CRQ) across five domains: linguistic accessibility, conceptual clarity, content appropriateness, technical structure, and cultural fit. Three independent Indonesian clinical psychologists rated each session (1–5 scale) and provided open-ended comments. Rater inclusion criteria were: licensed clinical psychologist, ≥3 years of clinical experience with sexual trauma cases. Percentages “4–5” reported in Results were calculated as the proportion of all item-ratings (reviewers × items) scoring 4 or 5, providing a session-level index of acceptability.

### 2.7 Measures

The Cultural Relevance Questionnaire (CRQ) was used as a structured tool to evaluate the cultural appropriateness and acceptability of the intervention modules across five domains: linguistic accessibility, conceptual clarity, content appropriateness, technical structure, and cultural fit. Each domain consisted of multiple items rated on a five-point Likert scale ranging from 1 (not appropriate) to 5 (highly appropriate).

In the present study, the CRQ was applied as a pragmatic evaluation tool within an intervention development framework, rather than as a fully standardized psychometric instrument. Given the early-phase nature of this research, the CRQ was used to generate structured expert feedback and to identify areas requiring refinement, rather than to establish definitive measurement properties such as reliability or construct validity across populations.

To enhance interpretability, domain scores were calculated as mean ratings across items and raters, and the proportion of ratings scoring 4–5 was used as an index of acceptability. In addition, qualitative feedback accompanying CRQ ratings was analysed thematically to complement quantitative findings and to ensure that cultural adaptation captured both semantic and contextual relevance.

Sensitivity analyses (leave-one-rater and leave-one-item) were conducted to examine the stability of domain scores. These analyses were intended to provide an indication of robustness within the constraints of a small expert sample, rather than formal reliability estimation.

### 2.8 Quantitative and qualitative analysis

Quantitative data from the CRQ were analysed using descriptive statistics, including mean scores, percentages, and levels of agreement, to provide an overall index of cultural fit. Cultural validation procedure and scoring. Three independent Indonesian clinical psychologists rated each session using the Cultural Relevance Questionnaire (CRQ; 1–5 Likert scale; five items per domain: linguistic accessibility, conceptual alignment, content appropriateness, technical structure, and cultural fit). The mean domain score was calculated as the average across all items within a domain, aggregated across raters. The percentage scoring 4–5 was computed as (number of item-ratings equal to 4 or 5) ÷ (total item-ratings = items × raters) × 100%. Item wording and examples are provided in Supplementary Table S3.

To assess robustness, we pre-specified leave-one-rater and leave-one-item analyses. Sensitivity analyses such as leave-one-rater and leave-one-item approaches are commonly used in small-sample validation studies to assess the stability and robustness of aggregated scores (Streiner et al., 2015). Effects were summarised as changes (*Δ*) in the mean domain score and in the percentage scoring 4–5. These sensitivity analyses were conducted to examine the stability of domain scores and to provide an indication of robustness within the constraints of a small expert sample, rather than serving as formal reliability estimation. Full outputs are reported in Supplementary Table S4.

Qualitative responses from the open-ended feedback were analysed thematically following Dey’s framework (Dey, 1993). Codes were organised into categories of functional, conceptual, and linguistic equivalence to ensure that cultural adaptation captured both semantic accuracy and contextual relevance.

### 2.9 Ethical considerations

Ethical approval was obtained from the Research Ethics Committee. All participants provided written informed consent prior to participation. They were fully informed about the study’s objectives, procedures, and their right to withdraw at any time without consequence. Anonymity was maintained by removing personal identifiers, and data were stored securely in accordance with institutional policy.

## 3 Results

### 3.1 Needs assessment

According to clinician’s reports, survivors frequently presented with overt symptoms, including freezing, mutism, crying spells, unstable affect, withdrawal from social interaction, and depressive complaints such as low mood and loss of interest. In parallel, covert difficulties were reported, including intrusive self-blame, shame, feelings of worthlessness, guilt, and anticipatory fear.

A consistent theme was the high prevalence of somatic symptoms, such as abdominal pain, headaches, nausea, palpitations, sweating, tremors, hot–cold flushes, disrupted sleep, and genital pain. These physical complaints often co-occurred with psychological symptoms, including anxiety, hypervigilance, flashbacks, suicidal ideation, and difficulties in trust and intimacy.

Beyond individual symptoms, psychologists emphasised systemic barriers: lack of supportive family or community environments, denial of survivors’ experiences, co-occurrence of poverty and dysfunctional family dynamics, and the added psychological strain of legal and forensic processes. Such contextual factors frequently contributed to high dropout rates, with survivors disengaging from care due to shame, distrust, avoidance, and dissociation.

Despite the predominance of body-based complaints, somatic methods were rarely applied in practice, as most interventions remained focused on cognitive or verbal approaches. Psychologists identified an urgent need for interventions targeting physiological dysregulation rather than relying exclusively on cognitive strategies. They recommended clear and simple psychoeducation on trauma physiology, strategies for safety and containment in group contexts, and gradual skill-building for body awareness, grounding, and emotional regulation.

To illustrate, psychologist notes survivors often used Indonesian expression such as “badan saya kaku” (my body feels stiff), “saya tidak bisa bicara” (I cannot speak), or “saya merasa hampa” (I feel empty) to describe their experience. These idioms of distress highlight how trauma was experienced and communicated through the body, reinforcing the needs for interventions that recognise both psychological and somatic dimensions of recovery.

The symptom profile and care barriers reported by clinicians are highly consonant with Indonesian cultural norms of hierarchy, collectivism, and emotional restraint. First, emotional restraint helps explain the prominence of somatic idioms (e.g., “*badan saya kaku*,” “*saya tidak bisa bicara*,” “*saya merasa hampa*”) and the covert manifestations of distress (shame, self-blame, anticipatory fear): when overt emotional expression is discouraged, suffering is more likely to be embodied and indirectly communicated, which can be misread in cognitively focused care. Second, hierarchy (high power distance) intersects with distrust, avoidance, and dropout: survivors may hesitate to challenge authority (including clinicians, family elders, or legal actors) or to disclose boundary violations directly, particularly when families deny their experiences. This dynamic supports the shift toward non-confrontational, body-based awareness of boundaries rather than immediate verbal disclosure. Third, collectivism frames recovery as relational: limited family/community support and stigma directly undermine regulation and engagement, while validating silence as participation and co-regulation in groups leverage cultural strengths to restore trust and safety.

This pattern is consistent with prior research indicating that trauma survivors in low- and middle-income countries frequently present with combined somatic and psychological symptoms (Devon E. Hinton and Patel, 2017; Miller and Rasmussen, 2014).

### 3.2 Intervention design

The programme structure was not adopted as a fixed standard protocol; rather, it was iteratively developed and culturally adapted based on findings from the needs assessment, expert consultation, and cultural validation, ensuring context-specific relevance for Indonesian survivors. The translation of these needs into a structured group intervention was guided by Somatic Experiencing®, a team of four SE® study experts affiliated with the SE International Institute and four SE® practitioners collaborated with the researcher. Their role was to ensure fidelity to core Somatic Experiencing® principles—simplicity, slowness, titration, and pendulation—while embedding flexibility for cultural adaptation.

The team proposed that early sessions should prioritise trust and safety building through introductions, group agreements, and reflection on participants’ safety needs, rather than immediate engagement in body-based practices. They also emphasised the use of gentle, choice-based language (“can” rather than “must”), e.g., “*kalau kamu mau, kamu boleh mencoba in*i.” (if you’d like, you can try this”), avoiding terms that may feel evaluative or triggering. The term body was flagged as potentially problematic for survivors of sexual abuse, with neutral alternatives (e.g., “*Apa yang kamu perhatikan di dalam dirimu atau di sekitarmu*?”) (“what do you notice inside or around you?”) recommended. Group norms were framed collaboratively as “*kesepakatan kelompok”* (group agreements), and safety check ins as “*kebutuhan rasa aman”* (safety needs).

Example facilitator prompts used in sessions: (1) “*Mari kita mulai dengan memperhatikan tiga hal yang kamu lihat di ruangan ini.”* (“Let us start by noticing three things you can see in the room.”); (2) “*Periksa: adakah bagian yang terasa sedikit lebih nyaman*?” (“Check: is there any part that feels even a little more comfortable?”); (3) “*Kalau dirasa terlalu banyak, kamu boleh berhenti dan kembali memperhatikan napas*.” (“If it feels like too much, you may pause and return to your breath.”)

### 3.3 Intervention goals and structure

Guided by the needs assessment and expert consultation, the intervention was designed to address domains identified as relevant to survivors’ experiences: including PTSD-related symptoms, resilience-related capacities, and quality of life. This domains were selected to inform future evaluation using instruments such as the PCL-5, CD-RISC-25C-25, and WHOQOL-BREF. A group format was selected for scalability and cost-effectiveness in a clinician-scarce context.

Programme structure. The programme comprised ten sessions (≈180 min each) for 10–12 participants. Every session had explicit objectives and 3–4 structured activities to minimise fatigue and support pacing. Sessions opened and closed with orienting practices. Core components included: (1) brief psychoeducation on trauma, stress, nervous system, delivered in short segments and plain Indonesian (e.g., membumi for grounding) to normalize somatic reactions; (2) graduated somatic skill development, sequenced from stabilisation-oriented practices (orientation, grounding/membumi, gentle movement) followed by felt sense, titration/pendulation, and discharge to respect norms of restraint while gradually expanding emotional capacity; (3) culturally aligned delivery and safety routines: choice-based language and neutral prompts avoid evaluative tones and power struggles inherent in hierarchical contexts; co created group agreements and structured turn-taking to protect confidentiality, share regulation, and prevent dominance/withdrawal; and boundary work (e.g., string boundary exercise and projecting voices exercise) via interoception provides a culturally safe alternative to direct confrontation. In sum, the clinicians’ observations map closely onto Indonesian value patterns, justifying an intervention that emphasises somatic literacy, relational safety, and gentle pacing over purely cognitive techniques. See Supplementary Table S2 Bilingual terminolongy glossary.

### 3.4 Safety and group protocols

Ensuring participant safety was identified as a central priority in both the needs assessment and expert design discussions. Survivors of sexual assault often enter group interventions with heightened sensitivity to boundaries, distrust of others, and vulnerability to emotional or physiological dysregulation. To address these concerns, the intervention incorporated multi-layered safety protocols and structured group processes.

Prior to the start of the intervention, participants received pre-group orientation materials that outlined the purpose of the sessions, expectations for participation, and available support. This preparatory step was designed to foster predictability and reduce anticipatory anxiety.

At the group level, explicit agreements were established in the opening session, including principles of confidentiality, respect, and mutual responsibility for emotional regulation. These agreements were revisited periodically to reinforce group cohesion and provide a collective framework for safety.

Facilitators received targeted training in rupture–repair strategies and co-regulation skills, equipping them to respond effectively to moments of participant distress or disengagement. Specific techniques included grounding through physical contact with external supports (e.g., feet on the floor, hands on the floor), use of orienting and titration to manage activation, and verbal reassurance delivered in culturally sensitive, non-directive language.

Each session was structured to open and close with orienting practices, allowing participants to enter and exit the therapeutic space within a regulated state. During sessions, brief pauses for grounding or reflective breathing were integrated after emotionally intense activities, ensuring that survivors remained within their window of tolerance.

These safety and group process protocols were designed to structure participation, support predictability, and provide multiple options for managing distress within sessions, while maintaining sensitivity to participants’ boundaries and cultural context.

### 3.5 Module review and cultural validation

Following development, the Indonesian-language modules underwent cultural validation. Quantitative analysis suggested strong cultural fit. Mean ratings were high across dimensions: linguistic clarity (*M* = 4.67, 93%), conceptual alignment (*M* = 4.46, 93%), content appropriateness (*M* = 4.43, 86%), technical structure (*M* = 4.56, 93%), and cultural resonance (*M* = 4.60, 90%). Overall, 90% of ratings fell between 4 and 5, indicating broad acceptability and robust cultural alignment of the modules. See Table 1 Cultural Relevance and Validity Testing (CRQ) for a summary.

**Table 1:** Cultural relevance and validity testing (CRQ).

Dimension	Mean Score	% Scoring	Interpretation
1. Linguistic	4.67	93%	Very strong – Clear and accessible language.
2. Conceptual	4.46	93%	Strong – Culturally meaningful therapeutic concepts.
3. Content	4.43	86%	Good – Minor improvements needed.
4. Technical	4.56	93%	Very strong – Practical and well-structured delivery.
5. Cultural Fit	4.60	90%	Excellent – Strong cultural resonance.

Sensitivity analyses. Robustness checks indicated *Δ* mean domain ≤ 0.05 and Δ percentage scoring 4–5 ≤ 2 percentage points in both leave-one-rater and leave-one-item analyses; domain interpretations (Good/Strong/Very strong/Excellent) were unchanged (see Supplementary Table S3).

### 3.6 Qualitative feedback

Qualitative feedback provided richer insights into module strengths and areas for refinement. Thematic analysis identified five key domains:

1. Linguistic and emotional accessibility – reviewers endorsed the overall compassionate and accessible language but noted that SE®-specific terms such as *grounding*, *felt sense*, and *pendulation* were unfamiliar to many survivors and English session titles were not easily understood, especially those with limited education. They recommended replacing these with intuitive Indonesian phrases (e.g., *merasakan sensasi tubuh* for felt sense or *membumi for grounding*) to ensure resonance. Session titles in English were also considered confusing and should be translated into Bahasa Indonesia.

*“Istilah grounding tidak mudah dipahami. Akan lebih kontekstual jika menggunakan kata membumi (‘mengakar/membumi’ dalam bahasa Indonesia”)* (“The term *grounding* is not easily understood. It would be more contextual to use *membumi* ‘to root/ground oneself’ in Indonesian”). — Reviewer 1.

“*Judul pertemuan perlu diterjemahkan ke bahasa Indonesia dan istilah spesifik perlu dijelaskan dengan bahasa awam” (“*The session title needs to be translated into Indonesian and specific terms need to be explained in layman’s language*”).* - Reviewer 2.

Indonesia value link: Hierarchy & emotional restraint: neutral, non-technical phrasing reduces status distance and pressure to display emotion.

2. Conceptual and cultural resonance – module content was seen as strongly aligned with survivors’ lived experiences, particularly emotions of shame, silence, and fawning in patriarchal contexts. Session 7, which addressed anger and shame, was described as especially relevant. They recommend keep shame-fawning focus; frame anger as protective energy and normalise non-disclosure pathway’s

“*Topik tentang rasa malu dan menjilat sangatlah tepat, karena emosi tersebut umum dialami oleh perempuan dalam konteks patriarki Indonesia”* (“The topics of shame and fawning are highly appropriate, as these emotions are commonly experienced by women in the Indonesian patriarchal context”). — Reviewer 3.

*“Bagi beberapa orang, merilis shame tidak harus ‘memberitahu dunia’; lebih penting merangkul diri”* (“For some, releasing shame does not have to be about ‘telling the world’; it’s more about embracing yourself”).– Reviewer 2.

Indonesia value link: Collectivism & emotional restraint: validating silence as participation and using somatic rather than confessional routes fits local norms.

3. Content and symbolic relevance – symbolic expressions were generally valued, but some metaphors (e.g., light in the heart) were considered too abstract unless paired with concrete explanations. The inclusion of dissociative symptoms (e.g., “living as if in a dream”) and their somatic correlates (e.g., freeze response) was praised as a strength, as these matched common survivor experiences. They also recommend give prompts specific and brief; explain tissue-blowing as supporting releasing shame/bravery.

*“Simbol membayangkan napas memenuhi hati dengan cahaya terlalu abstrak dan harus disederhanakan atau dijelaskan lebih lanjut.”* (“The symbol of imagining breath filling the heart with light is too abstract and should be simplified or explained further”). — Reviewer 2.

*“Projecting voice / boundary & safety sangat bermanfaat; jelaskan dinamika meniup tisu untuk releasing shame & empowering bravery” (*Projecting a good voice and security is very beneficial; explaining the dynamics of blowing a tissue to release shame and empower courage”). *-Reviewer 1.*

Indonesia value link: Hierarchy and collectivism: shared, observable actions create common meaning without personal disclosure.

4. Technical and practical fit – while the overall session structure was coherent, reviewers raised concerns regarding pacing, facilitator roles, and participant readiness. Session 5 was considered too dense, and session 6’s pelvic-focused grounding exercise was flagged as potentially triggering (adjusting pacing and activity load). Reviewers recommended adopt stabilisation-first sequencing, insert pause windows, provide safe alternatives, use small group for somatic exercises, specify co-facilitator role to monitor switches; include ball/tissue as concrete regulators.

*“Beberapa peserta mungkin belum siap untuk aktivitas yang berfokus pada tubuh bagian dalam. Pilihan adaptif dan penjelasan fasilitator tambahan diperlukan”* (“Some participants may not be ready for deep body-focused activities. Adaptive options and additional facilitator explanations are needed*”).—* Reviewer 1.

*“tools bola dapat membantu beralih dari kondisi tegang ke tenang, co-fasilitator memegang peran pada setiap kondisi peralihan, somatic practices butuh kelompok kecil (<4)”* (“ball tools can help transition from tense to calm states, co-facilitators play a role in each preservation state, somatic practices require small groups (<4)”). – Reviewer 3– Reviewer 3.

Indonesia value link: emotional restraint: titration/pendulation respect gradual engagement; Hierarchy: clear roles/turn-taking reduce dominance and withdrawal.

5. Challenges and recommendations – several refinements were suggested:

First, reviewers highlighted the importance of introducing trauma psychoeducation earlier in the intervention (Sessions 1–2), as delayed introduction may contribute to confusion and reduced engagement when participants encounter somatic experiences without sufficient conceptual understanding. Second, concerns regarding emotional overload during somatic practices led to the integration of structured grounding pauses and reflective breaks throughout sessions, ensuring that participants remain within a manageable window of tolerance. Third, reviewers emphasised the need for flexibility in session pacing, recognising that participants’ emotional readiness and somatic capacity may vary significantly, particularly in trauma-exposed populations. Fourth, the need to clarify the therapeutic purpose of specific somatic activities (e.g., tissue blowing, vocal projection) underscored the importance of explicitly linking each exercise to trauma recovery processes, rather than presenting them as abstract or symbolic tasks. Finally, reviewers cautioned against the use of generalised or externally oriented metaphors (e.g., “giving voice to the world”), recommending instead a focus on internal self-connection and self-acceptance, which better aligns with participants’ comfort levels and cultural norms.

Importantly, these challenges and recommendations were not treated as supplementary observations but served as central drivers of the cultural adaptation process (Table 2).

**Table 2:** Review feedback summary per module.

Session	Mean score	Strength identified	Suggested revisions
1	4.5	Safe group introduction, normalization of discomfort	Introduction trauma psychoeducation and clarify PTSD term
2	4.6	Culturally familiar body awareness activities	Add guidance for participants unfamiliar with introspection
3	4.5	Clear sensory tracking, suitable for trauma	Simplify abstract concepts and balance small group setup
4	4.6	Concrete psychoeducation	Add explanation trauma symptom in early sessions
5	4.3	Strong activities on boundaries and voice	Reduce activity load
6	4.4	Well-structured pendulation and containment	Provide alternative for grounding pelvic region
7	4.8	Deep relevance of shame, fawning, and patriarchy	Include emotional pause breaks between dense content
8	4.7	Effective somatic expression of aggression and shame	Avoid overly complex instructions
9	4.6	Gradual integration of SE processes good pacing	Clarify somatic intention, avoid English-only text
10	4.6	Balanced closure using internal-external focus	Avoid overtones, ensure cultural neutrality

Taken together, the findings across needs assessment, expert consultation, and cultural validation collectively informed iterative refinement of the intervention. Specifically, the prominence of somatic expressions of distress and identified care barriers shaped the prioritisation of stabilisation-oriented and body-based components in early sessions. Cultural validation feedback led to targeted modifications in language (e.g., use of non-technical Bahasa Indonesia), session pacing (e.g., inclusion of micro-pauses and reduced activity density), and delivery strategies (e.g., choice-based instructions, non-confrontational boundary work, and flexible participation norms). Furthermore, conceptual refinements deepened the focus on shame and fawning as protective responses relevant to the Indonesian patriarchal context. Additionally, reviewer input guided the adaptation of potentially sensitive practices such as providing non-pelvic grounding alternative and clarified the co-facilitators’ role in monitoring participant activation and using concrete tools (e.g., balls and tissues) for regulation. Overall, these refinements ensured that the intervention structure, content, and delivery remained closely aligned with the cultural, clinical, and contextual considerations identified throughout the development process (Table 3).

**Table 3:** How findings shaped the final protocol.

Finding (results/CRQ)	Adjustment in the final protocol
Prominent somatisation (palpitations, tremor, sweating, sleep problems, GI pain/tension)	Stabilisation-first sequencing (Sessions 1–3): graded sensory orienting; membumi micro-practices; end-of-session down-regulation (longer-exhale breathing, peripheral body scan); pendulation from discomfort → neutral areas; comfortable-range neck/shoulder micro-movements.
Shame, distrust, avoidance, dropout risk	Group agreements validating “silence as participation”; voluntary turn-taking; choice-based language throughout; opt-out at any time; safety check-ins at opening/closure.
Linguistic (CRQ): SE® terms unclear; English titles confusing	Indonesian glossary; intuitive phrasing replacing jargon; all session titles in Indonesian; simple cue cards for facilitators.
Conceptual (CRQ): relevance of shame/fawning; avoid confrontational narratives	Emphasise “protective energy” of anger; normalise non-disclosure pathways; replace abstract metaphors with observable actions.
Technical (CRQ): Session 5 too dense; pelvic focus sensitive; need breaks	Fewer activities in Session 5; 30–60-s micro-pauses per block; non-pelvic grounding options; small groups (<4) for somatic practice; co-facilitator monitors activation shifts.
Cultural fit (CRQ): hierarchy & emotional restraint	Non-evaluative instructions; no touch; structured turns; interoceptive boundary work rather than verbal confrontation.

These adaptations are directly reflected in the final intervention design, particularly in the emphasis on stabilisation-first sequencing, culturally sensitive language, structured pacing, and flexible participation norms.

## 4 Discussion

A systematic review and meta-analysis synthesized 16 empirical studies investigating SE in trauma-exposed populations (Kuhfuß et al., 2021). The pooled results indicated moderate effect sizes for PTSD symptom reduction (Hedges’ *g* ≈ 0.58) and somatic symptoms (Hedges’ *g* ≈ 0.61), alongside improvements in affect regulation and wellbeing. However, major methodological limitations were identified: only a minority of studies were randomized controlled trials, most relied on small convenience samples, non-standardized outcome measures, and high heterogeneity (I^2^ > 70%). Limited follow-up and therapist adherence documentation further constrain generalizability. Thus, while preliminary evidence supports SE’s potential to reduce trauma symptoms, rigorous research remains needed, especially in LMICs and collectivist societies.

SE operates through bottom-up regulation processes, focusing on bodily awareness (interoception), incremental engagement (titration), and oscillation between distress and safety (pendulation). These processes are theoretically grounded in models such as polyvagal theory, which propose that trauma recovery involves restoration of autonomic flexibility and social engagement (Payne et al., 2015; Porges, 2022). However, these mechanisms remain primarily theoretical frameworks, and direct empirical evidence linking these specific processes to clinical outcomes is still evolving (Kuhfuß et al., 2021). It is therefore important to distinguish between theoretical plausibility and empirical demonstration. Thus, references to autonomic regulation in this study should be understood as conceptual rationale guiding intervention design rather than empirically verified mechanisms within this study.

Importantly, this study focuses on cultural adaptation and validation rather than clinical effectiveness; therefore, findings should be interpreted as evidence of feasibility and contextual fit rather than therapeutic efficacy. Cultural validation supports acceptability and contextual fit, it does not in itself suggest therapeutic impact or symptom change, which require empirical testing in later phases. Accordingly, the present study adopts a developmental perspective, and its findings should be interpreted as evidence of feasibility and cultural relevance rather than efficacy. Given these limitations in the evidence base, our study was designed to specifically address gaps in context-specific adaptation and validation for collectivist LMICs.

This study developed and culturally adapted a group-based Somatic Experiencing® intervention for Indonesian women survivors of sexual assault with post-traumatic stress disorder (PTSD) symptoms, through systematic integration of survivor-informed needs, local clinical perspectives, and international SE® expertise. Our findings underscore three major contributions: (1) a detailed mapping of trauma-related somatic symptoms largely unaddressed by existing therapies; (2) the translation of these needs into a culturally resonant, body-oriented group intervention; and (3) robust cultural validation confirming acceptability across multiple dimensions.

The needs assessment revealed a high burden of somatic distress—including autonomic arousal (palpitations, sweating, tremors, hot–cold flushes), persistent somatisation (abdominal pain, headaches, nausea, disrupted sleep, genital pain), and psychological sequelae (shame, dissociation, intrusive self-blame, relational withdrawal) a pattern consistent with cross-cultural evidence showing that trauma in many settings is embodied and often under-addressed by conventional care (Devon E. Hinton and Patel, 2017; Miller and Rasmussen, 2014).

These findings were translated into a structured, culturally resonant intervention designed with fidelity to SE® principles while incorporating local norms of collectivism, hierarchy, and emotional restraint. This process aligns with established frameworks for cultural adaptation, which emphasise systematic modification of language, content, and delivery while maintaining core therapeutic principles (Bernal and Sáez-Santiago 2006; Castro et al., 2004). This approach accords with broader guidance on adapting psychological interventions to cultural and contextual realities (Payne et al., 2015).

Cultural validation then supported high acceptability across linguistic, conceptual, technical, and cultural domains (e.g., intuitive terminology, pacing adjustments, safety protocols), this convergence with prior work on culturally responsive trauma care supports the ecological fit of our adaptations (Hinton and Patel, 2017). Nevertheless, such validation reflects alignment with context and stakeholder perspectives, and should not be interpreted as evidence of intervention effectiveness or clinical benefit.

### 4.1 From findings to design decisions

Somatic prominence. Sessions 1–3 prioritise graded visual–auditory orienting, *membumi* micro-practices, and *down-regulation* before closure—in keeping with SE®‘s emphasis on titration and autonomic regulation (Payne et al., 2015). For autonomic complaints (palpitations, sweating, tremor), early sessions place sensory orienting and micro-grounding (feet–floor contact; labelling three neutral stimuli) ahead of other techniques to reduce sympathetic activation. Sleep disturbance is targeted at session end via longer-exhalation breathing, a peripheral *body scan* highlighting neutral/pleasant sensations, and “*good-enough*” cues rather than total relaxation—a sequence aligned with SE® protocols for safe de-activation (Payne et al., 2015). Gastrointestinal discomfort is addressed through pendulation from uncomfortable to neutral areas (e.g., palms, chair-back contact) and gentle *micro-movements* to facilitate discharge without premature interoceptive exposure. Headache and muscle tension are approached with environmental orienting and comfortable-range neck/shoulder *micro-movements*, punctuated by 30–60-s pauses to remain within the window of tolerance.

Systemic barriers and dropout. To reduce attrition, this study combined co-created group agreements that validate silence as legitimate participation, voluntary *turn-taking* to prevent dominance/withdrawal in hierarchical settings, and consistently *choice-based* language (“*if you’d like…*”). These mechanisms are congruent with evidence on high power-distance cultures and culturally attuned support processes, where predictability and agency foster trust and engagement (Hofstede et al., 2010; Kim et al., 2008).

Hierarchy and non-confrontation. Boundary work is interoceptive (string boundary, voice projection) rather than confessional, mapping onto Indonesian high power-distance norms and recommendations from culturally responsive psychotherapy (Hofstede et al., 2010; Hinton and Patel, 2017).

Terminology clarity. A bilingual glossary standardises *grounding → membumi*, *felt sense → merasakan sensasi tubuh*, and *pendulation → pendulasi/peralihan bertahap perhatian*; all session titles are in Indonesian with cue cards for facilitators—a strategy endorsed in culturally sensitive adaptations to reduce jargon and increase resonance (Hinton and Patel, 2017).

Session density and safety. Sessions 5–6 were lightened, micro-pauses scheduled, and culturally safe non-pelvic alternatives added for grounding—consistent with SE® guidance to avoid excess arousal and to privilege titrated exposure (Payne et al., 2015). A co-facilitator monitors activation shifts, leads micro-pauses every 15–20 min, and conducts post-session check-ins when delayed reactivation occurs.

CRQ-driven adjustments. Domain-specific refinements directly followed the CRQ: linguistic—glossary and translated titles; conceptual—emphasis on shame/*fawning* as protective energy and a non-disclosure pathway; technical—reduced load in Session 5, scheduled micro-pauses, small groups for somatic practice, explicit co-facilitator roles; cultural fit—no touch, non-pelvic alternatives, non-evaluative instruction. This tight coupling between feedback domains and protocol changes mirrors recommended adaptation workflows (Castro et al., 2004).

The intervention underwent rigorous cultural adaptation involving input from three independent expert reviewers. The use of the Cultural Relevance Questionnaire (CRQ) supported high acceptability across linguistic, conceptual, and symbolic domains. Reviewers highlighted the importance of adjusting SE-specific terminology (e.g., *“pendulation,” “felt sense”)* into more intuitive expressions, which aligns with prior findings indicating that local metaphors and culturally embedded symbols enhance engagement and therapeutic depth in trauma-focused interventions (Devon E. Hinton and Patel, 2017). Click or tap here to enter text. Click or tap here to enter text. Click or tap here to enter text. Click or tap here to enter text. Click or tap here to enter text.

Three foundational Indonesian cultural values—hierarchy, collectivism, and emotional restraint—were integrated as follows: *hierarchy*, by avoiding direct confrontation with authority; *collectivism*, by fostering shared presence and validating silence as participation; and *emotional restraint*, by using titration and pendulation to support gradual engagement (Hofstede et al., 2010; Kim et al., 2008; Payne et al., 2015). Consistency is reinforced by no-touch safety protocols, crisis-referral pathways, and an explicitly stated *opt-out* option during orientation—all aligned with best practice guidance in humanitarian MHPSS (Tol et al., 2011).

Our results accord with global evidence that survivors in low- and middle-income countries (LMICs) frequently present with somatic symptoms insufficiently treated by top-down cognitive approaches (Hinton and Lewis-Fernández, 2011; Miller and Rasmussen, 2014). Converging with emerging evidence on SE®‘s potential to reduce PTSD symptoms and improve regulation, our contribution is to culturally adapt and validate a *group* format in a low-resource setting (Kuhfuß et al., 2021; Winblad et al., 2018). By embedding SE® within Indonesian sociocultural frameworks—normalising silence as participation, framing progress as collective healing, and replacing catharsis with gradual titration—this study extends literature on culturally adapted trauma interventions (Castro et al., 2004; Hinton and Patel, 2017). The group format leverages collectivist values and peer support, consistent with meta-analytic and refugee-context evidence that social connectedness buffers PTSD (Nickerson et al., 2011; Ozer et al., 2003). Thus, the primary contribution is a culturally acceptable, implementation-ready model rather than a definitive claim of clinical effectiveness. Accordingly, the present findings should be interpreted as evidence of feasibility in terms of design and cultural alignment, rather than evidence of efficacy.

### 4.2 Strengths and limitations

Key strengths include the multi-layered methodology integrating local psychologists, SE® practitioners, and international experts; the use of a structured framework (McKenzie et al., 2021) to guide adaptation; and robust cultural validation through both quantitative (CRQ ratings) and qualitative methods. The systematic documentation of somatic distress and its translation into session content represents an important innovation for LMIC contexts. Several limitations should be acknowledged. First, the absence of national prevalence data on trauma-related somatic symptoms in Indonesia constrains comparisons with high-income settings and limits broader contextual interpretation. Second, survivor perspectives were not directly included in the intervention development phase and were incorporated indirectly through clinician reports, which may reduce ecological validity and limit the depth of lived-experience representation. Third, the involvement of Somatic Experiencing®-affiliated experts introduces the possibility of allegiance bias in both intervention development and evaluation. Fourth, the cultural validation relied on a small number of expert reviewers (*n* = 3), which may limit the robustness of the validation process, constrain generalizability, and potentially inflate agreement estimates. These imitations are consistent with early-phase intervention development. Future research should expand survivor involvement through participatory co-design approaches, include more diverse and independent stakeholders, and examine cross-cultural transferability of the intervention across different settings.

The proposed intervention structure (10 sessions of approximately 180 min) may also raise feasibility considerations, particularly in low-resource and time-constrained settings. While this format was designed to ensure adequate coverage of core components during the development phase, its practicality in real-world implementation remains to be established. Future feasibility and pilot testing will be essential to assess participant burden, engagement, and retention, and to inform potential adaptations such as shorter sessions, modular formats, or context-specific adjustments.

### 4.3 Implications for practice, policy, and research

The findings underscore the necessity of incorporating body-based and culturally adapted approaches into trauma services in Indonesia and similar LMICs. For clinical practice, the adapted SE® intervention provides a replicable model for addressing both somatic and psychological dimensions of PTSD, moving beyond cognitive-heavy modalities. At the policy level, integration into community mental health and gender-based violence services could help close treatment gaps, particularly in contexts where professional resources are scarce. For research, the study provides a framework for documenting cultural relevance systematically and highlights the value of mixed method approaches in intervention development.

### 4.4 Future directions

Future studies should build on this foundation by conducting fully powered randomized controlled trials (RCTs) to evaluate feasibility, pilot, clinical effectiveness and mechanisms of change. Longitudinal research is needed to examine durability of gains in PTSD symptoms, resilience, and quality of life. Cross-cultural comparative studies could explore adaptation processes in other collectivist or LMIC settings, contributing to the global literature on somatic trauma therapies. In addition, participatory research with survivors should be prioritised to refine language, metaphors, and delivery strategies. More broadly, this work contributes to international calls for trauma interventions that are context-sensitive, body-oriented, and scalable in resource-limited environments (Patel et al., 2018; Tol et al., 2011).

## 5 Conclusion

This study presents constitutes Phase 1 of a broader research program and presents the first systematically developed and culturally validated group-based Somatic Experiencing® intervention for Indonesian women survivors of sexual assault. By explicitly addressing the gap between survivors’ high burden of somatic distress and the limited availability of body-oriented approaches in LMICs, the intervention provides a novel and contextually grounded model of trauma care.

The development process combined multi-layered expert input, survivor-informed needs assessment, and rigorous cultural validation, resulting in high acceptability across linguistic, conceptual, technical, and cultural dimensions. The integration of collectivist values, hierarchy sensitivity, and emotional restraint into session design ensured ecological validity and enhanced therapeutic resonance.

These findings highlight the promise of culturally adapted, group-based somatic interventions as feasible, safe, and socially resonant approaches for trauma recovery in resource-constrained, collectivist societies. While further research is required to evaluate to evaluate feasibility, pilot, clinical effectiveness, mechanisms of change, and scalability, this work establishes a strong foundation for expanding body-oriented trauma therapies in LMIC settings where conventional top-down approaches remain insufficient.

## Data Availability

The datasets presented in this study can be found in online repositories. The names of the repository/repositories and accession number(s) can be found in the article/Supplementary material.
