# Supplementary material for: Cultural development and validation of a group-based Somatic Experiencing® intervention for Indonesian women survivors of sexual assault with PTSD symptoms: a mixed-methods study
Source: Front Psychol. 2026 May 21;17:1751747. doi: 10.3389/fpsyg.2026.1751747 (PMC13234763; doi:10.3389/fpsyg.2026.1751747)
Supplement: Supplementary file 1 [file Supplementary_File_1.docx]

Supplementary Material

# Table S1. Program continuity illustrating the sequential design linking Phase 1 (development & cultural validation) to Phases 2–3 (feasibility and effectiveness evaluation).

| **Phase** | **Aim / Focus** | **Design & Participants** | **Main Measures / Analyses** | **Expected Outputs** |
| --- | --- | --- | --- | --- |
| Phase 1: Development & Cultural Validation (Present Study) | Develop and culturally validate the SE® group intervention for Indonesian women survivors of sexual assault. | Mixed-method, cross-sectional design; expert psychologists and SE® practitioners. | Cultural Relevance Questionnaire (CRQ)**;** thematic analysis of qualitative feedback. | Final validated intervention modules; evidence of linguistic, conceptual, and cultural fit. |
| Phase 2: Feasibility & Pilot (ISRCTN 58257113) | Assess feasibility, acceptability, and preliminary clinical trends. | Two-arm pilot RCT; women 18–45 with sexual-assault history and PTSD symptoms. | Feasibility metrics (recruitment, retention, adherence); SRS, HAT; PCL-5, CD-RISC-25, WHOQOL-BREF. | Feasibility confirmed; pilot effect sizes; refinement of procedures for full RCT. |
| Phase 3: Effectiveness Evaluation | Evaluate clinical effectiveness of the SE® group intervention. | Parallel-group RCT (n = 207; 2:1 allocation). | PCL-5, CD-RISC-25, WHOQOL-BREF; SPSS v22.0 quantitative & qualitative thematic analysis. | Effectiveness and mechanism evidence; model for trauma-informed intervention in LMICs. |

# Table. S2. Bilingual terminolongy glossary

| **English term** | **Indonesian rendering** | **Notes for facilitators** |
| --- | --- | --- |
| Grounding | *Membumi* | Gunakan contoh konkret: “rasakan telapak kaki menyentuh lantai.” |
| Orienting | *Orientasi* | “Perhatikan 3 hal yang kamu lihat/dengar/rasakan saat ini.” |
| Felt sense | *Merasakan sensasi tubuh* | Hindari kata “tubuh” jika sensitif → gunakan “di dalam dirimu/di sekitarmu.” |
| Titration | *Titrasi (pendosingan kecil) | Analogi: bayangkan segelas H₂O bening lalu ditetesi sirup pekat setetes demi setetes, diaduk perlahan, cek rasa setiap tetes dan berhenti sebelum terlalu manis. Demikian juga di sesi: paparkan sensasi/emosi sulit setetes (≤10–20 detik), kembali ke sumber nyaman, ulangi bila aman. Hindari “tuang sekaligus” (paparan besar) yang bisa melampaui *window of tolerance*. |
| Pendulation | *Pendulasi / peralihan bertahap perhatian* | Pergi–pulang antara area/tema yang nyaman ↔ kurang nyaman, dalam dosis kecil. |
| Choice-based language | *Bahasa berbasis pilihan* | “Kalau kamu mau… kamu boleh mencoba…” |
| Discharging | *Pelepasan (energi/tegangan) | Proses alami ketika sistem saraf melepas muatan setelah titrasi/pendulasi: hela napas panjang, menguap, hangat, aliran air liur, getar halus pada tangan/kaki, ingin meregang. Tidak dipaksa, tidak dikejar, cukup diberi ruang & waktu; hentikan jika terasa terlalu kuat dan arahkan kembali ke *membumi/orientasi*. Normalisasikan dengan kalimat: “Kalau ada napas panjang/bergetar sedikit, itu tanda tubuhmu sedang merilis ketegangan.” |

# Table S3. Cultural Relevance Questionnaire (CRQ): domains, items, and examples

| **Domain** | **Item ID** | **Item wording (English)** | **Example mapping to modules** |
| --- | --- | --- | --- |
| **Linguistic accessibility** | L1 | Wording is clear and free of jargon. | Sessions 1–2: “safe group introduction” |
|  | L2 | Key terms are defined and used consistently across sessions. | Glossary (e.g., *grounding* → *membumi*; *felt sense* → “sensing”) |
|  | L3 | Instructions are concise and understandable across education levels. | Sessions 3–4: sensory tracking |
|  | L4 | Translations preserve the intended meaning. | CRQ: Linguistic clarity M = 4.67 |
|  | L5 | Choice-based phrasing is used (e.g., “If you’d like…”). | Safety language throughout |
| **Conceptual alignment** | C1 | Therapeutic concepts fit survivors’ lived experiences. | Session 7: shame/anger focus |
|  | C2 | Shame–*fawning* can be framed as protective energy where appropriate. | Session 7 |
|  | C3 | A non-disclosure pathway is normalised. | Session 1: group agreements |
|  | C4 | Somatic skills are linked to psychological targets. | Down-regulation for sleep; boundaries/voice in Session 5 |
|  | C5 | Sequencing reflects titration/pendulation logic. | Stabilisation-first (Sessions 1–3) |
| **Content appropriateness** | A1 | Activities are feasible in community settings. | Duration options (90–180 min) |
|  | A2 | Metaphors are concrete or clearly explained. | Simplify “light in the heart” metaphor |
|  | A3 | The activity load in Session 5 is manageable. | Reduce tasks; add micro-pauses |
|  | A4 | Alternatives are provided for sensitive content. | Non-pelvic grounding in Session 6 |
|  | A5 | Each practice includes safety notes. | Safety prompts across sessions |
| **Technical structure** | T1 | Session objectives and flow are explicit. | Appendix A sessions 1–10 |
|  | T2 | Micro-pauses are scheduled (every 15–20 min). | Sessions 5–7 pacing |
|  | T3 | The co-facilitator role is specified. | Monitor activation shifts |
|  | T4 | Guidance on group size and turn-taking is clear. | Small groups <4 for somatic practice |
|  | T5 | Materials are minimal, low-cost, locally available. | Balls/tissues as regulators |
| **Cultural fit** | F1 | A no-touch policy and respectful distance are explicit. | Safety protocol |
|  | F2 | Non-pelvic grounding alternatives are offered. | Session 6 revision |
|  | F3 | Silence is validated as participation. | Group agreements, Session 1 |
|  | F4 | A non-evaluative tone avoids power struggles. | Choice-based instructions |
|  | F5 | Examples and language reflect Indonesian norms. | Local idioms (e.g., “my body feels stiff”) |

# Table S4. Sensitivity-analysis outputs (CRQ)

| **Domain** | **Full mean** | **Full % 4–5** | **Max \|Δ\| mean (leave-one-rater)** | **Max \|Δ\| % (leave-one-rater)** | **Max \|Δ\| mean (leave-one-item)** | **Max \|Δ\| % (leave-one-item)** | **Interpretation unchanged?** |
| --- | --- | --- | --- | --- | --- | --- | --- |
| Linguistic clarity | 4.67 | 93.0 | ≤0.05 | ≤2.0 | ≤0.04 | ≤1.5 | Yes |
| Conceptual alignment | 4.46 | 93.0 | ≤0.05 | ≤2.0 | ≤0.04 | ≤1.5 | Yes |
| Content appropriateness | 4.43 | 86.0 | ≤0.05 | ≤2.0 | ≤0.04 | ≤1.5 | Yes |
| Technical structure | 4.56 | 93.0 | ≤0.05 | ≤2.0 | ≤0.04 | ≤1.5 | Yes |
| Cultural fit | 4.60 | 90.0 | ≤0.05 | ≤2.0 | ≤0.04 | ≤1.5 | Yes |
